# Analytical Extraction Methods and Sorbents’ Development for Simultaneous Determination of Organophosphorus Pesticides’ Residues in Food and Water Samples: A Review

**DOI:** 10.3390/molecules26185495

**Published:** 2021-09-10

**Authors:** Krishna Veni Veloo, Nur Amirah Syahirah Ibrahim

**Affiliations:** 1 Faculty of Agro-Based Industry, Jeli Campus, Universiti Malaysia Kelantan, Jeli 17600, Kelantan, Malaysia; 2 Faculty of Bioengineering and Technology, Jeli Campus, Universiti Malaysia Kelantan, Jeli 17600, Kelantan, Malaysia; amirahsyahirah323@gmail.com

**Keywords:** extraction, pesticides, sample preparation

## Abstract

Extensive use of organophosphorus pesticides in agriculture leads to adverse effects to the environment and human health. Sample preparation is compulsory to enrich target analytes prior to detection as they often exist at trace levels and this step is critical as it determines the concentration of pollutants present in samples. The selection of a suitable extraction method is of great importance. The analytical performance of the extraction methods is influenced by the selection of sorbents as sorbents play a vital role in the sensitivity and selectivity of an analytical method. To date, numerous sorbent materials have been developed to cater to the needs of selective and sensitive pesticides’ detection. Comprehensive details pertaining to extraction methods, developed sorbents, and analytical performance are provided. This review intended to provide a general overview on different extraction techniques and sorbents that have been developed in the last 10 years for organophosphorus pesticides’ determinations in food and water samples.

## 1. Introduction

Pesticides are compounds applied on crops to eliminate or control pests, rodents, fungi, and weeds [1]. They are also used in agriculture to increase yield and prolong the shelf life of crops [2]. The class of organophosphorus pesticides (OPPs) has been extensively used worldwide. A study conducted by Li et al. [3] revealed that the class of OPPs account for 72% of the total amount of pesticides used in China. The extensive use of OPPs in agriculture might be probably due to their inexpensive cost and efficiency [4].

However, critical adverse effects may arise from the uncontrolled use of OPPs in the agriculture industry, which could lead to soil, water, and crop produce contamination [5] and affects human health [2]. Therefore, demand for a stringent monitoring on OPPs’ residue in food and the environment has been increasing [6]. Therefore, OPPs’ residue determination has become a primary concern and is crucial for food and environmental control.

Nevertheless, direct determination of OPPs’ residues in complex matrices is a challenging task, owing to differences in polarities, solubility, pKa values, and volatilities and analysis accuracy is greatly affected due to the presence of matrix interferences as the OPPs often present at trace level concentration [7]. Hence, the sample preparation step, which consists of sample enrichment and cleanup, is essential to eliminate matrix interferences prior to detection. The sample preparation technique is also important to preconcentrate the analytes. This step is the bottleneck of the analytical procedure as it plays a crucial role in ensuring the samples are free from matrix interferences and are amenable for analysis using analytical instruments. 

Multiple analytical methods such as liquid-liquid extraction (LLE), solid-phase microextraction (SPME), stir-bar sorptive extraction (SBSE), and solid-phase extraction (SPE) have been adopted for OPPs’ analysis, which has been widely reported in the literature [8,9,10]. These techniques have their own inherent advantages and drawbacks, which prompted the development of new analytical methods, particularly the microextraction method, which offers notable merits in terms of facile extraction procedure, being less time consuming, minimizing organic solvents’ consumption, and enhancing extraction efficiency [11]. In addition, the possibility of reusing the extraction device is an important advantage of microextraction techniques.

Recently, development of selective sorbents has been emerging and received substantial interest due to its crucial roles in enhancing selectivity and sensitivity of analytical methods. Therefore, numerous adsorbents have been developed and reported in the literature. Numerous review articles have been published in recent years on the advances of extraction techniques and sorbents’ development for pesticides analysis. Over 150 articles were found when a search in the Science Direct was carried out using the keywords “sorbents”, “extraction techniques”, “food and water samples”, and “organophosphorus pesticides” within a period of 2011–2021. In 2018, a review published by Samsidar et al. [12] in Trends in Food Science & Technology covered a review of extraction and analytical and advanced methods for determination of pesticides in environment and foodstuff. However, the review did not provide an extensive discussion on the sorbents used in the extraction methods.

This present paper briefly discusses the method performance and strengths and weaknesses of sample preparation techniques that are commonly used for determination of OPPs. In addition, a short review on the development of extraction sorbents is also highlighted. This review presents an overview on how the selection of the extraction method and the choice or sorbents influences the performance of a developed method. 

## 2. Organophosphorus Pesticides

**Figure 1:**
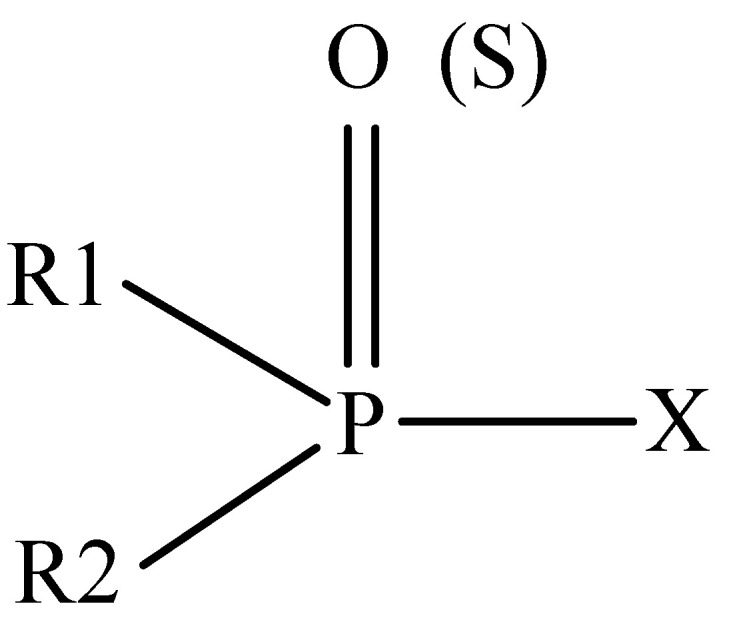
General structure of organophosphorus pesticides [14].

Classifications of pesticides are based on origin, target organism, and chemical structure. There are two major groups of pesticides, which are chemical pesticides and bio-pesticides. Pesticides can also be categorized based on chemical structure such as organochlorine pesticides (OCPs), organophosphorus pesticides (OPPs), carbamates, and pyrethroids [12,13]. In this paper, only OPPs will be briefly discussed, as this review paper is focusing on the extraction methods and sorbents’ development for determination of OPPs in food and water samples. Figure 1 shows the general structure of OPPs. R1 and R2 represent alkyl-, alkoxy-, alkylthio-, or amino groups. X denotes the acyl residue (labile fluorine-, cyano-, substituted or branched aliphatic, aromatic, or heterocyclic groups) [14].

OPPs have been used as plasticizers, stabilizers in lubricating and hydraulic oils, flame retardants, and gasoline additives [15]. OPPs have also been used for medical usage to treat myasthenia gravis and glaucoma [16]. OPPs are extensively used worldwide in agriculture for crop protection [17], as they are the most effective and compelling method to protect plants from pests and have improved crop yields and agricultural productivity. However, critical adverse effects may arise from the uncontrolled use of OPPs in the agriculture industry, which could lead to soil, water, and crop produce contamination [5] and affect human health. The physical properties and toxicity class of several OPPs are presented in Table 1. OPPs are considered non-persistent (shorter half-life) due to rapid degradation in the environment [17].

OPPs are neurotoxins (nerve poison), by which they inactivate cholinesterase enzyme and disrupt the transmission of nerve impulses [19]. Consequently, a wider and stringent residues’ monitoring has gained upswing demand from consumers regarding the environment and food contaminated by OPPs [6]. Therefore, OPPs’ residue determination has become a primary focus and is crucial for food and environmental control.

The risk of possible adverse effect of OPPs on human health prompted authorities to set a guideline on permissible quantity of OPPs’ residue to ensure agricultural products are safe for consumption. Regulatory authorities such as the World Health Organization (WHO), European Union (EU), and Codex Alimentarius Commission (CAC) have established the OPPs’ maximum residue level (MRL) of agricultural products for consumer reference. The MRL was established to ensure food commodities are safe for human consumption. The European Union indicated that water for human consumption should not exceed 0.1 µg L^−1^ for each pesticide and 0.5 µg L^−1^ for total pesticides [20]. Table 2 shows the maximum residue limit (MRL) of several OPPs in fruits commonly consumed by consumer as given by the EU Regulations (EC) No. 2020/1085, No. 2017/978, and No. 2015/399 [21,22,23].

## 3. Sample Preparation Techniques and Development of Extraction Sorbents

The selection of sample preparation technique is of utmost important to ensure that accurate and precise analysis of targeted analytes can be achieved. Different sample matrices require different sample preparation techniques. Highly complex matrices such as biofluids (i.e., blood, plasma, and urine) require a highly selective method to obtain high sensitivity and robustness for the analysis of small molecules due to the presence of endogenous (e.g., metabolites of the target analyte, proteins, or lipids) or exogenous (substances introduced during sample processing and analysis) compounds [24]. Fruits and vegetables contain carotenoids, carbohydrates, and fat, which also require selective extraction to eliminate the matrix interference. Therefore, consideration on the choice of sample preparation technique is vital. This paper will be focusing on the sample preparation techniques for food and water samples. 

The OPPs’ determination in food samples is challenging due to the presence of matrix interference in complex matrices and low analytes’ concentration. Therefore, development of a facile and sensitive detection method is crucial for determination of OPPs [25]. Sample preparation must also be fast, easy, simple, low-cost, and amenable to a broad range of analytical instruments. 

The accuracy and precision of an analytical method is highly dependent on selection of sample preparation techniques [26]. Over the past few years, numerous extraction techniques for determination of OPPs in food and environmental samples have been reported. Trends in sample preparation for pesticides’ residues have been focusing toward miniaturization of analytical methods and development of selective sorbents. Extraction techniques can be categorized into two categories, which are liquid-based extraction and solid-based extraction. 

### 3.1. Liquid-Based Extraction 

Liquid-liquid extraction (LLE) is a conventional extraction technique that involves partitioning between two immiscible phases. LLE offers several advantages, including high-separation factors and high-purity products. However, there are several shortcomings associated with LLE, including being time-consuming, needing a large volume of solvent consumption, being tedious, and formation of emulsion, which could potentially impose health and environmental safety threats [27,28]. 

Therefore, recent studies have been primarily centered on miniaturization of LLE to develop facile, quick, and environmentally and economically friendly extraction methods. Hence, miniaturized techniques such as liquid-liquid microextraction (LLME), dispersive liquid-liquid microextraction (DLLME), vortex-assisted liquid-liquid microextraction (VALLME), and single-drop microextraction (SDME) have been developed. LLE required a large volume of sample to obtain low LOD. Thus, modification in LLE has been carried out particularly in terms of organic phase volume reduction into microliters [29,30,31]. Table 3 shows the summary of past studies on the application of LLE as sample preparation technique for OPPs’ determinations.

DLLME was introduced by Rezaee et al. [32]. Since then, DLLME has been widely used for the extraction of various compounds in liquid samples. In DLLME, an extractant is formed as small droplets of the water-immiscible organic solvent in the sample solution by using water-miscible organic solvent [29]. Zhao et al. [33] developed a miniaturized LLE technique known as dispersive liquid-liquid microextraction (DLLME) for the extraction of OPPs from cucumber and watermelon samples prior to gas chromatography flame photometric detection (GC-FPD). The developed method showed satisfactory recovery (67–111%) for all the targeted analytes. A comparative study was performed with conventional LLE to assess the applicability and analytical performance of the developed DLLME method. Results indicated that both DLLME and conventional LLE methods exhibited comparable analytical performance in terms of recovery and precision. Nonetheless, the DLLME method exhibited lower LOD (0.1–0.19 ng g^−1^) in comparison to conventional LLE (0.8–2.0 ng g^−1^). This is because DLLME achieved a higher enrichment factor than conventional LLE. Besides that, the developed DLLME showed advantages over the conventional LLE, by which the volume of organic solvent was substantially reduced and evaporation procedure was eliminated, which, in turn, reduced the analytical process time. 

However, DLLME possesses major shortcomings in terms of the limitation of solvents’ selection, capable of forming a dispersive phase and that primarily consisted of chlorinated solvents [34]. Therefore, a new analytical method was introduced by Psillakis et al. in 2010 [30,35], termed as vortex-assisted liquid-liquid microextraction (VALLME) to overcome the drawback of DLLME method. Zacharis et al. [31] developed a VALLME method for determination of 12 selected OPPs from water and wine samples using gas chromatography-mass spectrometry (GC-MS). The method exhibited low LOD (2–11 ng L^−1^) with good enrichment factors ranging from 65–389. Besides that, satisfactory recoveries (70–120%) were achieved for all the targeted OPPs. The method provides advantages in terms of ease of operation, high enrichment factor, good analytical performance, cost reduction, and eliminating the use of additional dispersing solvents. This could also probably be due to the use of GC-MS as the analytical method since GC is a good tool for the analysis of volatile and easily vaporized compounds. Besides that, MS provides high sensitivity and accuracy, better anti-interference effect, and good reproducibility [12].

Recently, a new green extraction technique, termed as supramolecular solvent-based microextraction (SUPRAS), has received significant interest due to its inherent properties, particularly in mitigating the adverse effects of organic solvents to the environment [30]. SUPRAS corresponds to water-immiscible solvents consisting of amphiphile aggregates, which are generated via self-assembly of amphiphilic molecules. These solvents are excellent alternative solvents to the conventional solvents for the preconcentration of pesticides residues using microextraction techniques as they provide various interactions such as hydrogen bonding, ionic bonding and hydrophobic interaction [36,37,38].

A nanostructured supramolecular solvent-based LLME method for preconcentration of methyl parathion from water samples coupled with high-performance liquid chromatography (HPLC) was developed by de Oliveira et al. [30]. Under optimized conditions, the method showed low LOD (0.27 ng mL^−1^), which was probably due to its good enrichment factor (139). Besides that, the SUPRAS method exhibited excellent recovery, ranging from 92–109.9%, with excellent RSD (2.23–3.6%). The good analytical performance was due to its capability to extract polar and non-polar analytes simultaneously, as the SUPRAS has diverse regions of polarity as a result of its ordered structures. Besides that, this method has replaced the use of hazardous organic solvents, which are commonly used in conventional methods, with non-toxic amphiphilic compounds as extractant solvent. This makes the SUPRAS method more environmentally friendly. 

In conclusion, many improvements and developments have been carried out in LLE to overcome its drawbacks and limitations. However, the application of LLE-based techniques has significant limitations in terms of sensitivity by which sorbent-based techniques such as solid-phase extraction (SPE), solid-phase microextraction (SPME), and stir-bar sorptive extraction (SBSE) offer lower LOD in comparison to LLE-based techniques. This might probably be due to the combination of physical properties (sorbent porosity and surface area) and chemical properties (molecular interaction via functional moieties) of the sorbents that play crucial roles in enhancing the extraction efficiency of targeted analytes. 

Nonetheless, the sensitivity of solvent-based extraction techniques could be enhanced by exploring the potential of supramolecular solvents due to its amphiphilic properties. Besides that, SUPRAS is environmentally friendly (non-volatile and inflammable). These favorable characteristics make SUPRAS microextraction technique a promising alternative to the conventional liquid-based extraction techniques.

### 3.2. Solid-Based Extraction

#### 3.2.1. Solid-Phase Microextraction (SPME)

Solid-phase microextraction (SPME) is an extraction technique introduced by Pawliszyn and Arthur in 1989 [41]. It has shown tremendous growth as an alternative to other extraction techniques due to its principle as it incorporates sampling, extraction, enrichment, and sample introduction into a single step without solvent. The extraction occurs via sorption of the targeted compound from the sample matrix onto the sorbent coating followed by desorption of the analyte from the coating into an appropriate instrumental system via thermal (headspace-GC) or solvent (HPLC or capillary electrophoretic analysis) [42,43]. 

SPME has demonstrated significant impacts in a solvent-free procedure, by which it excludes the use of organic solvents, miniaturization, and automation in the sample preparation field [44]. Besides that, it reduces the analysis time by incorporating sampling, extraction, enrichment, and sample introduction into one step. SPME is also feasible to be combined with various analytical instruments such as gas chromatography (GC), high-performance liquid chromatography (HPLC), and capillary electrophoresis (CE) [45,46,47]. Despite the numbers of advantages in comparison to other methods, SPME possesses several disadvantages such as limited sorbent loading, which could affect its sensitivity, fragility, and degradation of the coated fibers, instability of the coating in organic solvent, and time consuming to achieve equilibrium. Another significant disadvantage is a limited number of sorbent coatings available commercially, which, in turn, makes the morphology and chemistry of the coatings restricted [41,42]. Besides that, the recoveries are generally low as SPME is a non-exhaustive extraction technique [48].

A summary of past studies on the application of SPME as a sample preparation technique for OPPs’ determinations is presented in Table 4. Rodrigues et al. [49] developed a method for the preconcentration of OPPs in cows’ milk using SPME in headspace mode (HS-SPME) and polydimethylsiloxane/divinylbenzene (PDMS/DVB) as sorbent. The method showed LOD ranging from 2.16–10.85 ng mL^−1^ for all the targeted analytes. The recoveries of the developed method that were exhibited were comparable to and even higher than the previous reported studies, except for ethion, due to its high hydrophobicity (Log K_ow_ = 5.07), by which it has higher affinity toward the sample matrix that is richer in lipids than the sorbent [50]. The method is capable of detecting OPPs in cows’ milk exposed to OPPs below their limits of quantification. 

Numerous types of sorbent coatings have been developed to improve extraction efficiency via modification of coating materials. Carbon nanotubes (CNTs), graphene, and metals are commonly used for the modification process [50,51]. As a result, this led to the emergence of modified nanomaterials. However, despite the high extraction efficiency of the modified nanomaterials, several shortcomings were identified, which are associated with tedious preparation procedures and expensive precursors [52]. Therefore, Saraji et al. [52] prepared a modified halloysite nanotube (MHNTs) as SPME coating for OPPs’ enrichment in water, cucumber, and apple samples prior to (gas chromatography-corona discharge ion mobility spectrometry (GC-CD-IMS) analysis. MHNTs were selected due to structure similarity with CNTs and being non-toxic and environmentally friendly in comparison to CNTs. The developed technique obtained low LOD (0.01–0.03 ng mL^−1^), indicating its sensitivity toward OPPs. Besides that, good recovery (84–97%) and good precision (3–9%) were achieved for all real samples. The good analytical performance of SPME-MHTs could possibly be due to high surface area of MHTs (102 m^2^ g^−1^) and molecular interaction between the targeted analytes and MHTs via hydrophobic and polar interaction (NH_2_ and OH groups). Therefore, the properties enhanced the extraction capability of the sorbent toward polar and semi-polar analytes. In recent times, metal-organic frameworks (MOFs) have also been used as SPME sorbent. MOFs has been getting much attention due to their pronounced physicochemical properties such as high surface areas, being porous, and having tunable pore size, functionality, and thermal stability, which make it a promising SPME coating material [52]. Many studies have reported the application of MOFs as SPME sorbent coating. However, Wu et al. [53] and Chaikittisilp et al. [54] reported that some MOFs’ structures collapsed when exposed to high temperatures. This restricts their application in SPME. Therefore, Pang et al. [55] prepared a nitrogen-doped metal organic framework (MOF)-based porous carbon (C-(C_3_N_4_@MOF)) as a new SPME coating for determination of 14 selected OPPs in five fruits’ samples coupled with GC-MS. The sorbent material was prepared via carbonation of a graphitic carbon nitride (g-C_3_N_4_)-templated MOF (NH_2_-MIL-125).

The incorporation of g-C_3_N_4_ in MOF led to the generation of a porous carbon structure, forbid agglomeration, and collapse of pores during pyrolysis [56,57]. 

The developed method exhibited low LOD (0.23 to 7.5 ng g^−1^) and good recovery, ranging from 82.6–118%. Results indicated that the C-(C_3_N_4_@MOF) method showed good sensitivity toward polar OPPs. This probably was due to the existence of nitrogen, which increased the polarity of the carbon material [58]. The extraction efficiency of the method was also promoted by the presence of π-stacking and hydrophobic interactions between the sorbent and OPPs as a result of the presence of graphitic sp2-hybridised carbons, C=O, and O-C=O in the C-(C_3_N_4_@MOF). In addition, the prepared coating material could be used over 100× without substantial loss of extraction efficiency. This indicates that the sorbent coating possessed good durability and reusability.

To date, numerous studies have been reported to overcome the disadvantages of SPME, especially in terms of coating materials. Many modifications on coating materials have been introduced as an effort to enhance the extraction efficiency of SPME coating sorbent. However, in general, the recoveries of targeted analytes were still low despite the low LODs. This could probably be because sorbent coatings were lacking in thermal stability, by which they were susceptible to degradation when exposed to high temperature during the desorption process. Hence, further improvement needs to be carried out, particularly in terms of coating material that possesses high thermal stability, which is capable to endure SPME conditions since it is the key factor in achieving successful extraction of target analytes.

#### 3.2.2. Stir-Bar Sorptive Extraction (SBSE)

Stir-bar sorptive extraction (SBSE) is an extraction technique involving partitioning of target analytes between a liquid sample and a stationary section-coated stir bar, by which the SBSE device is directly introduced into an aqueous sample. It was designed to overcome the extraction capacity limitation and fragile coatings in SPME by providing larger, solid-phase volumes [61,62]. SBSE possesses several advantages over SPME, especially in sensitivity and accuracy for trace level analysis in complex matrices. It is effective for extraction of dilute and low-concentration samples [62,63]. 

SBSE has several shortcomings including its incapability to desorb extracted analytes directly into the GC injection port. An additional procedure is required to desorb the analytes into an appropriate solvent, which could affect its sensitivity [63]. Furthermore, commercial, alternative, sorbent materials to polydimethylsiloxane (PDMS) are limited [43]. SBSE presents low recovery for highly polar analytes due to weak hydrophobic interaction owing to the non-polar nature of PDMS [61]. 

Therefore, there has been a considerable interest among researchers to address the shortcomings of SBSE method. Table 5 shows the summary of past studies on the application of SBSE as a sample preparation technique for OPPs’ determinations. The development of SBSE method is primarily focused on the sorbent coating. To date, studies are limited on the application of SBSE, due to limited variety of stir-bar coatings since only PDMS is commercially available. Hence, a new sorbent coating has been prepared by using PDMS in combination with other materials that are commercially available such as polyvinyl alcohol, graphene, and multiwalled carbon nanotubes (MWCNTs) to enhance the extraction performance of SBSE [64,65,66]. 

A new coating for SBSE is necessary to increase the extraction efficiency of OPPs since OPPs consists of a wide range of polarity. Therefore, Yu and Hu [66] prepared a new sol-gel PDMS/PVA-coated stir bar for the preconcentration of five selected OPPs from honey samples prior to GC-FPD analysis. The new PDMS/PVA-coated stir bar exhibited a large surface area (21.248 m^2^ g^−1^), which was 500× higher than the commercial PDMS coating. It also showed good regeneration and stability, by which it can be used 50× without substantial decrease in the extraction efficiency of OPPs. Besides that, the method obtained good LOD (0.013–0.081 ng mL^−1^) and good recovery (81–124%), which indicate its sensitivity and accuracy.

Next, OH-functionalized multiwalled carbon nanotubes (OH-MWCNTs) were prepared by Ahmadkhaniha and Rastkari [65] as SBSE stir-bar coating for OPPs’ extraction from water samples coupled with GC-MS. Previously, they had prepared MWCNTs as SBSE coating material for OPPs’ and polycyclic aromatic hydrocarbons’ (PAHs) determination. However, there were several drawbacks such as coating instability and lacking in sensitivity [67]. This could have been due to insolubility of CNTs in organic and aqueous solvents, which restricts its usage in a sol-gel preparation. Therefore, OH-MWCNTs were used to enhance CNTs’ dispersion in a sol-gel system. The OH-MWCNTs were selected as they enhance CNTs’ solubility and generate stable structure, as this polymer binds the MWCNTs’ stationary phase to the growing silica network during polycondensation process [65,68]. The developed method employing the OH-MWCNTs’ sorbent and GC-MS showed low LOD (5–10 ng L^−1^) and satisfactory RSD (7.2–12.4%). These results showed the sensitivity and validity of the method for the extraction of OPPs in water samples. Nevertheless, the method showed adequate recovery for the targeted analytes, ranging from 67–84%. This indicated that the method was lacking in accuracy.

Jafari et al. [64] developed zirconium dioxide-reduced graphene oxide (ZrO_2_-rGO) as a new coating for SBSE. The ZrO_2_-rGO sorbent coating was used for enrichment of ethion from water samples prior to NCD-IMS analysis. The LOD of the method was 1.5 ng mL^−1^ with good RSD (6%). Besides that, high recovery was also obtained, ranging from 93–97%, indicating the accuracy of the developed method. The good analytical performance was possibly due to a high surface area of graphene and good sensitivity of ZrO_2_ toward phosphate groups in ethion, which promoted better extraction efficiency.

The applicability of SBSE was further improved and extended into microextraction following the trend of method miniaturization. Benedé et al. [69] introduced a new method, known as stir-bar sorptive-dispersive microextraction (SBSDµE). This method incorporates the principles of two extraction methods, namely, stir-bar sorptive extraction (SBSE) and dispersive solid-phase microextraction based on magnetic nanoparticles (d-µSPE). In SBSDµE method, magnetic nanoparticles (MNPs) were coated on a magnetic stir bar. At a low stirring rate, the technique acts as SBSE, while at a high stirring rate, dispersion of MNPs into a sample solution (or desorbing solvent) took place. The MNPs return to the magnetic bar once the stirring process ended.

Therefore, Madej et al. [70] developed a SBSDµE method with magnetically modified graphene (G-Fe_3_O_4_) for the preconcentration of seven different classes of pesticides from water samples coupled with HPLC. The performance of the developed method was compared with magnetic solid-phase extraction (MSPE). Comparable recoveries of targeted analytes were achieved, ranging from 20–75% and 22–82% for SBSDµE and MSPE, respectively. However, the developed SBSDµE method offers a rapid extraction process and easy separation of sorbent from the sample solution. The method could also provide a high enrichment factor due to effective mixing of a large volume of samples, hence, improving the LOD.

Based on Table 3, the SBSE methods obtained satisfactory extraction recoveries despite the low LODs. Thus, further improvement and development should be carried out to improve their extraction performances in terms of coating material and technical aspect. However, previous studies concentrated on the modification of coating material and only limited studies reported on the improvement of structural modification of stir-bar devices [71]. Therefore, more studies should be carried out on enhancing stir-bar devices and automation of SBSE. 

#### 3.2.3. Solid-Phase Extraction (SPE)

Solid-phase extraction (SPE) involves two different phases partitioning where compounds of interest are retained between a solid phase (sorbent) and a liquid phase (sample) [74]. SPE process comprises four steps, which are column conditioning, sample loading, washing, and elution. The procedures are pertinent despite the types of sorbents chosen, formats (cartridge, disks, 96-well plate, and pipette tip), and the operational mode, whether it is automated (online SPE) or conventional procedure (off-line SPE).

The ability to enrich trace compounds simultaneously, along with matrix interferences’ elimination; rapid, easy operation; low organic solvents’ consumption; and the ability to provide a high preconcentration factor, make SPE superior to other sample preparation techniques [75]. In short, SPE is widely used and a reliable sample preparation technique. Therefore, these advantages have attracted significant interests among researchers, which led to substantial development of SPE.

To date, numerous SPE methods have been developed using various materials including graphene [76], magnetic nanocomposites [77], and mesoporous silica doped with titanium [78]. A summary of past studies on the application of SPE as a sample preparation technique for OPPs’ determinations is shown in Table 6. Nevertheless, some sorbents suffer from several shortcomings including high values of LOD, low extraction recovery, and incapability to simultaneously extract large number of OPPs. 

C_18_-functionalised Fe_3_O_4_@mSiO_2_ microsphere was prepared by Xie et al. [79] for enrichment of selected OPPs from water samples. The SPE method showed high LOD for the targeted analytes, ranging from 1.8–5.0 µg L^−1,^ which exceeded the MRLs for drinking water (0.1 µg L^−1^) as indicated by the European Union Directive. Hence, numerous SPE sorbent materials have been developed to enhance extraction efficiency and selectivity, primarily in terms of structures and functionalities. This is because the primary aspect of SPE depends on the sorbent phase as it controls the extraction performance and sensitivity and precision of analysis [42]. Therefore, consideration on the physicochemical properties of sorbent and target analytes should be taken into account during method development to promote good adsorbent–adsorbate interaction [80].

To date, molecularly imprinted polymers (MIP) have become a notable approach for selective SPE, in which it is based on molecular recognition mechanisms. The MIPs possess recognition sites that are complementary in shape (structure) and physicochemical properties with the target analyte (template molecule) [81]. Hence, the selectivity of MIPs has initiated the extensive use of MIPs as SPE selective sorbent for determination of OPPs, as eloquently stated by Boulanouar et al. [82]. Besides that, MIP-based sorbents are also stable with organic solvents, extreme pH, and high temperatures [76]. However, MIPs’ primary drawback is their incapability to simultaneously extract huge number of OPPs due to the wide range of structures and polarities. Hence, different sorbents need to be developed for different analytes [80].

A solid-phase extraction-capillary electrophoresis (SPE-CE) method was developed by Zhao et al. [83] using MIP (trichlorfon as template) for the extraction of trichlorfon from cucumber, lettuce, and radish samples. The LOD of the developed method was 4.9 ng g^−1^ with satisfactory recovery ranging from 77.6–93.2%. It was noted that the method is lacking in sensitivity, as indicated by the high value of LOD in comparison to other methods. This probably is due to the use of CE, as its main limitation is sensitivity of detection, owing to its small inner diameter of the capillary (50–75 μm) [12].

The lack in sensitivity of MIP-SPE sorbents initiated attempts to improve the sensitivity by combining MIPs with other materials. He et al. [84] prepared a new SPE sorbent from a combination of MIP and restricted access material (RAM). Malathion was used as a template molecule and glycidilmethacrylate (GMA) as a pro-hydrophilic co-monomer for determination of six selected OPPs from honey samples prior to quantification by GC-FPD. MIPs are best known for their high selectivity due to specific recognition capability for targeted analytes. However, MIPs are incapable of eliminating macromolecules such as proteins and lipids simultaneously. This is due to strong adsorption of the macromolecules to the MIPs’ surfaces via hydrophobic interaction, which affects the recognition properties of MIPs [85]. RAM is a porous support, which is capable of eliminating macromolecules based on the size-exclusion mechanism [86]. Therefore, it is interesting to combine MIPs and RAM for selective enrichment of targeted OPPs. 

The developed method showed low LOD for all the selected OPPs, ranging from 0.5–1.9 ng mL^−1^ with excellent RSD (2.25–5.12%). Comparison was carried out with several sorbents, namely, MISPE, C_18_, and Florisil in terms of extraction recovery to determine the applicability of the developed method for honey samples. Results showed that the RAM-MISPE method exhibited high extraction efficiency (90.9–97.6%) in comparison to MISPE (90.5–96.2%), C_18_ SPE (80.1–85.9%), and Florisil SPE (72.3–77.4%), respectively. The developed method obtained high recovery due to its high selectivity toward the OPPs and good restricted access function. The good performance of the method is also probably due to the use of GC-FPD as a detection system, which is selective toward compounds containing sulfur and phosphorus such as OPPs. There was a minute difference in the extraction recovery between RAM-MISPE and MISPE, which was only 1%. The extraction recovery of C_18_ SPE and Florisil SPE were slightly lower in comparison to RAM-MISPE and MISPE, which were probably due to lack of affinities and non-specific recognition toward the analytes. In addition, the analytical time was significantly reduced, as the sample pre-treatment procedure for honey prior to extraction was omitted.

Over the past few years, attention has been primarily focused on the development of selective sorbent material for SPE. Immunosorbents (IS), also known as immunoaffinity extraction, has been introduced to cater to the need of selective support in sample preparation, which is based on the antigen–antibody interactions. Immunosorbents are synthesized by linking the antibodies to solid support. IS are highly specific for a particular analyte. However, they can also bind with structurally similar analytes, known as the cross reactivity of antibody [87].

The application of ISs for organophosphorus pesticides has been studied by Xu et al. [88]. The study revealed that 13 OPPs were successfully extracted using an in-house monoclonal antibody immobilised on CNBr-activated Sephrose 4B from a water sample with adequate recovery (60.2–107.1%) and low limit of quantification (0.01–0.13 ng mL^−1^). This work justifies that ISs are capable of simultaneous extraction of OPPs. The advantage of using IS is that the extraction and isolation of target compounds from complex matrices could be performed in one step, thereby eliminating the co-extraction of interferences problem due to a high degree of antibodies’ selectivity [88]. However, despite the high degree of selectivity shown for specific analytes, IS is not a promising sorbent in environmental analysis. This is because IS requires high cost for its production, involves tedious preparation, is time consuming, and has less availability and low capacity [89]. Identifying suitable antibodies for the sorbent is the main difficulty [86]. Besides that, it is probably due to the fact that there are multiclasses of pollutants differing in structures and polarities. Carbon-based nanomaterials such as fullerenes, graphene, and carbon nanotubes (CNTs) are commonly used for analytical purposes, and these materials have demonstrated their capabilities as excellent SPE sorbent due to giant π-electronic structure [87,88]. Recently, a graphene-based sorbent has prompted significant interest due to its promising advantages such as a high surface area, high adsorption capacity, it could be easily functionalized, and its facile preparation in comparison to CNTs [75,90].

A study conducted by Han et al. [76] reported that the SPE method developed using graphene as sorbent coupled with GC-MS showed good LODs (0.04–0.35 ng mL^−1^) and satisfactory recoveries, ranging from 69.8 to 106.2% for determination of OPPs in apple juices. However, several drawbacks of graphene such as strong hydrophobicity, π-π stacking, and van der Waals forces led to restacking and aggregation between graphene sheets, which resulted in the reduction of graphene surface area and, consequently, decrease in adsorption efficiency. Thus, graphene is not a practical SPE sorbent [91,92]. Therefore, modification of graphene has been initiated to alleviate this issue, by which graphene oxide and three-dimensional (3D) graphene that possess higher surface area and more polar moieties (i.e., hydroxyl and carbonyl groups) were developed [93,94]. 

Sun et al. [8] successfully enriched OPPs in water samples using SPE cartridge packed with 3D graphene aerogel prior to GC-MS analysis, and achieved low detection limits (LODs), ranging from 0.12–0.58 ng mL^−1^ with excellent recoveries (93.8–104.2%) for all targeted analytes. Hence, these studies indicated that a graphene-based sorbent is a promising material for the extraction of OPPs from different matrices.

Silica-based materials such as methyltrimethoxysilane, tetraethoxysilane, and polydimethylsiloxane have been used as sol-gel precursors to synthesise sorbent materials. The sol-gel technique is commonly used to prepare SPME- and SBSE-coating fiber for OPPs’ determination. As of 2011, the application of a sol-gel technique for the preparation of SPE sorbent was limited. However, Wan Aini et al. [20] introduced a novel organic-inorganic sol-gel hybrid MTMOS-TEOS as SPE sorbent for the extraction of OPPs from water and fruits’ samples prior to detection using GC-MS. The developed method showed low LOD (0.5–0.9 pg mL^−1^) and high recovery toward the targeted analytes because of the sol-gel hybrid mesoporous nature and high surface area. Besides that, the good performance of the method was also probably due to the use of GC-MS as the detection system. This is because GC-MS provides higher sensitivity and possesses good anti-interference effect and better accuracy. The sorbent demonstrated good stability and regeneration by which it could be regenerated 25 times without a significant decrease in extraction efficiency. As such, this study provides an additional insight into the potential to explore more hybrid materials prepared via sol-gel reaction for application in analytical extraction. Consequently, this study has prompted a trend in development of SPE sorbent using a sol-gel technique.

Several studies have reported the development of silica-based SPE sorbent using a sol-gel technique for OPPs’ determination [10,20,95]. A hybrid silica-based SPE sorbent methyltrimethoxysilane-cyanopropyltriethoxysilane (MTMOS-CNPrTEOS) was prepared by Wan Ibrahim et al. [95] via sol-gel technique for simultaneous extraction of polar (dicrotophos and methamidophos) and non-polar (diazinon, malathion, methidathion, and chlorpyrifos) OPPs from tap water and lake water samples prior to quantification using GC-MS. A commercial C_18_ SPE sorbent was used for comparison purpose. Results indicated that the developed method exhibited a low LOD (0.01–0.02 ng mL^−1^), which was 5–10× lower than the MRL set by the European Union (EU) for water samples. The LOD of the developed method was 5–8× lower in comparison to the LOD of C_18_ sorbent. The SPE-MTMOS-CNPrTEOS-GC-MS method showed good recovery, ranging from 84–99%, and excellent precision (0.06–6.5%). The remarkable results of the developed method could probably be due to the hybrid sorbent possessing high surface area (585 m^2^ g^−1^) and porous structure, which promotes the extraction efficiency. The sol-gel hybrid MTMOS-CNPrTEOS showed high selectivity toward the polar analytes compared to the non-polar analytes. These findings showed that the introduction of CNPrTEOS to MTMOS improved the hydrophilicity of the sorbent and promoted the interaction of both polar and non-polar OPPs with the developed sorbent. 

Magnetic nanoparticles (MNPs) and graphene were introduced in combination with silica-based materials via sol-gel technology to improve ease of separation, sorbent surface area, and enhancement of sorbent ability for simultaneous enrichment of polar and non-polar analytes. A study carried out by Nodeh et al. [10] successfully prepared magnetic nanoparticles’ graphene-based cyanopropyltriethoxysilane (Fe_3_O_4_@G-CNPrTEOS). The sorbent material showed efficient OPPs’ extraction from fresh cows’ milk samples with minimal sample preparation, by which it only involved simple dilution and omitted acid treatment for fat and protein matrix elimination. A gas chromatography micro-electron capture detector (GC-µECD) was employed as a detection system. The sorbent exhibited high tolerance to matrix interferences and good reusability (10 adsorption-elution cycles). It also showed high enrichment factors (2400) and sensitivity (0.01–0.6 ng mL^−1^). The developed method provided good recovery (82–94%), probably due to graphene hydrophobic properties and the presence of polar cyanopropyl moieties (-C≡N) from CNPrTEOS that enabled simultaneous preconcentration of polar and non-polar OPPs. Therefore, this study revealed that the fabrication of graphene- and silica-based materials shows excellent preconcentration of trace analytes from complex matrix.

Veloo and Ibrahim [96] developed a SPE method using sol-gel hybrid MTMOS-CPTES for the preconcentration of three selected OPPs, namely, chlorpyrifos, profenofos, and malathion from red apple and purple grape samples prior to GC-MS analysis. The developed SPE-MTMOS CPTES-GC-MS method achieved good LODs, ranging from 0.01–0.07 µg mL^−1^, with good recoveries (88.33–120.7%) for all the targeted analytes. The method used a low sample volume (1 mL) and eluent volume (1 mL) but the developed method was capable of providing a good detection limit. This indicates that the method provides merits in terms of reducing the sample size, decreasing organic solvents’ consumption, and needing shorter analytical time without affecting the sensitivity of the method. 

In recent times, miniaturization has become a trend due to various advantages over conventional extraction techniques including shorter extraction time, minimal sample volume, less consumption of organic solvents, and a facile extraction procedure. In 2003, Michelangelo & Lehotay [97] introduced a miniaturised SPE technique termed as dispersive-SPE (dSPE) for the extraction of pesticide residues in produce. Since then, dSPE has become a trend for the preconcentration of various analytes. dSPE involves the dispersion of sorbent material in a sample solution, which greatly increases the surface area in contact. Hence, the extraction efficiency was enhanced substantially.

Fakhari and Aladaghlo [98] developed a new method, known as solvent-assisted dSPE, for determination of OPPs. In this method, the dispersion of a sorbent into the aqueous sample maximised the interaction surface between the analytes and the sorbent. Next, centrifugation was carried out and the retained OPPs were dissolved in ethanol. The method exhibited low LOD (0.3 ng mL^−1^) and a pronounced enrichment factor, ranging from 368–376. The developed method offered merits in terms of portability, shorter analysis time, being simple, and minimizing cost. 

In conclusion, numerous studies have reported on the development of SPE sorbent from various materials. These studies revealed that the extraction efficiency of targeted analytes is greatly influenced by the properties of the sorbent. Therefore, the selection of sorbent material and the physicochemical properties of the targeted analytes should be taken into consideration. Besides that, the findings from the studies suggested that the properties of sorbents can be exploited to enhance trace analytes’ preconcentration. Therefore, there is a potential that a multiple ligand sorbent (i.e., bifunctional sorbent) bearing multiple functional moieties such as amino, cyano, and chloro groups can be developed via sol-gel reaction.

In the sample preparation field, it would be practical if a single SPE sorbent is capable of simultaneously preconcentrating all OPPs, since OPPs consist of a broad range of polarities and structures. Therefore, the development of functionalised materials as SPE sorbents, focusing on enhancement in selectivity, will continue to progress. Besides that, current research has also been focusing on miniaturization of extraction technique, which could mitigate the weaknesses of the conventional SPE techniques. 

A new research trend on the advances of SPE has also been noticed. Studies conducted by Amiri et al. [99] and Amiri and Ghaemi [100] introduced the application of sorbent coated on a stainless steel for the enrichment of OPPs and polyaromatic hydrocarbons. A high amount of coating was coated on the meshes, which resulted in the significant increase of surface area in contact. Consequently, this approach shortened the adsorption and desorption process. Besides that, the studies reported low LOD and high recovery of targeted analytes. Therefore, the results indicated that the combination of an improved extraction device and sorbent could result in synergistic effects that greatly improve the extraction efficiency of target analytes. Hence, the approach on enhancing the extraction efficiency via the advancement of SPE device (i.e., cartridge, frits) is a good strategy, other than focusing on the development of sorbent materials. Table 7 shows the summary of the advantages and limitations of sample preparation techniques for OPPs’ determination.

## 4. Overview on the OPPs’ Extraction Sorbents 

Class OPPs consists of diverse compounds, differing in structures and with a wide range of polarities [100]. Therefore, considerable attention must be given when selecting the extraction technique and sorbent material. It would be best if a sorbent material possesses both hydrophobic and hydrophilic properties so that huge numbers of OPPs’ compounds with different physicochemical properties could be enriched simultaneously. This approach could help in minimizing analytical process steps, reduce solvents’ consumption, save time, and reduce analysis cost.

Numerous sorbents have been developed to improve selectivity of extraction such as immunosorbents (IS), RAM, and MIP. IS, which is based on molecular recognition, have been used in pharmaceutical analysis, while RAM is efficient, particularly in eliminating huge interferences, while entrapping small molecules [79,80,86]. MIP is highly selective but only limited to the compounds with similar structures [100]. Graphene offers a huge surface area, which is a favourable sorbent property, as it could promote efficient extraction [98]. However, the use of graphene alone would lead to a decrease in the extraction efficiency due to aggregation of graphene sheets [89]. 

Hybrid silica sorbents prepared via sol-gel technology provide notable extraction performance. This could probably be due to the enhanced properties (i.e., thermal, mechanical, chemical stability) of the sorbent as a result of the combination of organic and inorganic precursors. The hybrid sorbents possessed a high surface area and were porous in nature [20,95]. Besides that, they are capable to extract both polar and non-polar simultaneously, due to the presence of bi-functional properties. The applications, advantages, and limitations of the sorbents are detailed in Table 8.

## 5. Conclusions and Future Outlook 

Sample preparation is a critical step in the development of an analytical method for trace analysis, as it influences the sensitivity and extraction efficiency of an analytical method. However, a conventional sample preparation step such as LLE and SPE usually involves high consumption of organic solvents and is tedious and time consuming. Therefore, this led to the emergence of miniaturized sample preparation methods to overcome the drawbacks of the conventional sample preparation methods. This trend could probably be associated with the current trends of the green chemistry approach. Miniaturised extraction techniques such as micro-dispersive solid-phase extraction, magnetic micro solid-phase extraction, liquid-phase microextraction, and vortex-assisted dispersive liquid-liquid microextraction have been receiving huge interest over the past few years. These techniques were developed to minimise organic solvents’ consumption, shorten the extraction time, and minimize costs. 

Next, the analytical performance of a sorbent-based extraction technique is greatly influenced by the selection of sorbent and sorbent synthesis technique. The interaction between the sorbent, analytes, and sample matrix needs to be considered to ensure good selectivity and extraction efficiency can be achieved. Previous studies reported that neat materials were found to be lacking in extraction efficiency. Therefore, modification of neat sorbent materials with other appropriate materials has become a common practice to alleviate the drawbacks. Modification is achieved via functionalization of a sorbent surface, using numerous materials such as the functionalisation of graphene and multi-walled carbon nanotubes with hydroxyl group, which has improved the sorbent extraction performance and selectivity substantially.

In recent times, hybrid materials are becoming a new trend in the development of sorbent materials. Hybrid materials exhibited remarkable improvement in materials’ properties and eliminated the disadvantages of neat materials. Numerous studies have reported the remarkable extraction performance of hybrid materials, which have substantially enhanced the selectivity toward target analytes and sensitivity of a developed method. Nevertheless, the sorbent preparation technique is also a crucial factor in determining the properties of a sorbent. Studies have been primarily centered on the functionalisation of sorbent materials via sol-gel technology, as it offers a facile sorbent preparation method with prominent sorbent properties. Sol-gel technique is often used to produce multiple functional ligands’ sorbent, which could significantly enhance sorbents’ selectivity. For example, amino and cyano moieties can be incorporated onto a single sorbent. The inherent properties make sol-gel technology an outstanding tool to prepare highly selective sorbents. Besides that, the desisgnable structure and tunable properties of sorbents via sol-gel technology could be a great help in developing a versatile extraction material and device. The combination of a good extraction sorbent and device could lead to synergistic effects on an efficient extraction process and simplifying the analytical protocols without affecting the sensitivity of an analytical method. 

In the future, the sorbents should focus on a single sorbent that possesses multiple ligands, which could extract diverse classes of pesticides simultaneously. Therefore, focus should be given on functionalisation of sorbent material. In this way, an analytical procedure could be reduced and minimise analysis time. Next, research should be carried out to develop a sorbent that possesses high stability and reusability. Therefore, this could effectively minimise analysis cost as the sorbent could be used repeatedly.

To date, numerous studies have been concentrating on the development of sorbents to enhance analytes’ extraction efficiency. However, a study on the extraction device was limited. Only several studies focusing on the improvement of the extraction device were reported. Therefore, it would be a good effort if more works would aim at focusing not only on improving the sorbent materials but also at developing extraction devices and analytical methods. This should be particularly targeted toward the automation of the extraction process and detection system, which could shorten the analytical process, improve the sensitivity, and eliminate tedious steps.

## Figures and Tables

**Table 1:** Physical properties and toxicity class of organophosphorus pesticides [18].

Pesticides	K_OW_	M.W	Action	Toxicity Class WHO
Cadusafos	3.90	270.40	Nematicide	1b
Diazinon	3.30	304.30	Insecticide	2
Chlorpyrifos-methyl	4.24	322.50	Insecticide	U
Malathion	2.75	330.40	Insecticide	3
Chlorpyrifos	4.70	350.60	Insecticide	2
Phenthoate	3.69	320.40	Insecticide	2
Profenofos	4.44	373.6	Insecticide	2
Ethion	4.28	384.5	Acaricide	2

**Table 2:** Maximum residue level (MRL) of OPPs in fruits (European Union (EU) Regulations (EC) No. 2020/1085, No. 2017/978, and No. 2015/399).

OPPs	MRL (mg/kg)	
	Apple	Grape
Chlorpyrifos	0.01	0.01
Profenofos	0.01	0.01
Malathion	0.02	0.02

**Table 3:** Summary of past studies on the application of LLE as a sample preparation technique for OPPs’ determinations.

Year	Technique	Analyte	Matrix	LOD	Recovery (%)	Analytical Technique	Ref.
2020	Nanostructured supramolecular solvent-based LLME	Methyl parathion	Water	0.27 ng mL^−1^	92–109.9	HPLC-UV	[30]
2020	Deep eutectic solvent-based ultrasound-assisted LLME	Phosalone and chlorpyrifos	Red grape juice and sour cherry juice	0.070–0.09 ng mL^−1^	87.3–116.7	HPLC-UV	[29]
2016	Miniaturised counter current LLE	Diazinon and malathion	Water	0.1 ng mL^−1^	96–110	GC-FID	[39]
2012	Vortex-assisted LLME	12 OPPs	Water and wines	2–11 ng L^−1^	70–120	GC-MS	[31]
2009	Dispersive liquid-liquid microextraction	Phorate, diazinon, disolfotane, methyl parathion, ethion, sumithion, malathion, fenthion, profenphose, phosalone	Tea	0.03–1 ng g^−1^	83.3–117.4	GC-FPD	[40]
2007	Dispersive liquid-liquid microextraction	Ethoprophos, parathion methyl, fenitrothion, malathion, chlorpyrifos profenofos	Cucumber and watermelon	0.01–0.19 ng g^−1^	67–111	GC-FPD	[33]

**Table 4:** Summary of past studies on the applications of SPME as a sample preparation technique for OPPs’ determinations.

Year	Sorbent	Analyte	Matrix	LOD	Recovery (%)	Analytical Technique	Ref.
2020	Nitrogen-doped porous carbon (C-(C_3_N_4_@MOF))	14 OPPs	Fruits and vegetables	0.23–7.5 ng g^−1^	82.6–118	GC-MS	[55]
2019	Polydimethylsiloxane/divinylbenzene (PDMS/DVB)	Parathion-ethyl, parathion-methyl, diazinon, chlorpyrifos	Strawberry jam	0.11–0.42 ng kg^−1^	N.A	MDGC-MS	[59]
2018	Poly(4-nitroaniline)/ poly(vinyl alcohol) electrospun nanofiber	Diazinon and chlorpyrifos	Water, orange juice and lemon juice	0.4–0.6 ng L^−1^	82–102	GC-CD-IMS	[14]
2017	Modified halloysite nanotubes (MHNTs)	Diazinon, parathion and fenthion	Water, cucumber and apple	0.01–0.03 ng mL^−1^	84–97	GC-CD-IMS	[52]
2015	Fe_3_O_4_/graphene nanocomposite	Diazinon, fenitrothion and malathion	Water	0.11–0.16 ng L^−1^	85–94	GC-FID	[50]
2014	Polydimethylsiloxane (PDMS)	Butachlor and chlorpyrifos	Urine	0.088–0.53 ng mL^−1^	83.06–99	GC-ECD	[60]
2011	Polydimethylsiloxane/ divinylbenzene (PDMS/DVB)	10 OPPs	Cow milk	2.16–10.85 ng mL^−1^	N.A	GC-MS	[49]

**Table 5:** Summary of past studies on the application of SBSE as a sample preparation technique for OPPs’ determinations.

Year	Sorbent	Analyte	Matrix	LOD	Recovery (%)	Analytical Technique	Ref.
2019	Magnetically modified graphene (G-F_3_O_4_)	Chlorpyrifos	Water	14 ng mL^−1^	44	HPLC-UV	[70]
2018	Zirconium dioxide-reduced grapheme oxide (ZrO_2_-rGO)	Ethion	Water	1.5 ng mL^−1^	93–97	NCD-IMS	[64]
2016	Polydimethylsiloxane (PDMS)	Profenofos, leptophos, isofenphos, prothiofos and methamidophos	Water	0.072–0.091 ng mL^−1^	82–94	^31^P QNMR	[72]
2016	OH-functionalised multi-walled carbon nanotubes (OH-MWCNTs)	Dichlorvos, diazinon, parathion-m and fenitrothion	Water	5–10 ng L^−1^	67–84	GC-MS	[65]
2013	Polydimethylsiloxane/polythiopene (PDMS/PTH)	Quinalphos, phorate, malathion, parathion, and fenitrothion	Water	0.011–0.038 ng mL^−1^	77–119	GC-FPD	[73]
2009	Polydimethylsiloxane/polyvinyl alcohol (PDMS/PVA)	Phorate, fenitrothion, malathion, parathion, and quinalphos	Honey	0.013–0.081 ng mL^−1^	81–124	GC-FPD	[66]

**Table 6:** Summary of past studies on the application of SPE as a sample preparation technique for OPPs’ determinations.

Year	Sorbent	Analyte	Matrix	LOD	Recovery (%)	Analytical Technique	Ref.
2020	MTMOS-CPTES	Chlorpyrifos, profenofos, malathion	Apple and grape	0.01–0.07 µg g^−1^	88.33–120	GC-MS	[96]
2019	MNPC based on Zn/Co-MOFs	Phorate, diazinon, malathion, fenthion, ethion	Apple, grape, pear, tomato, green jujube	0.018–0.045 ng g^−1^	84–116	GC-FPD	[101]
2018	MIP (monocrotopos as template and 3-aminopropyltriethoxysilane as monomer	Dimethoate, malathion, diazinon, methidathion, fenthion sulfoxide, fenitrothion, fenthion sulfone, pirimiphos-methyl fenthion and chlorpyrifos-ethyl	Almond oil	0.12–0.46 ng g^−1^	100–114	LC-MS	[82]
2017	Polymethacrylate-based sorbent modified with MNPs	Chlorpyrifos, phosmet and pirimiphos-methyl	Water	0.01–0.25 ng mL^−1^	71–98	LC-UV	[102]
2017	Fe_3_O_4_/CNT	Fenitrothion, ethion, profenofos	Water	0.097–0.124 ng mL^−1^	60–92	HPLC-UV	[77]
2016	Cyanopropyltriethoxysilane (CNPrTEOS)	Dicrotophos, diazinon, chlorpyrifos.	Water	0.072–0.091 ng mL^−1^	80.1–92.1	HPLC-UV	[103]
2015	RAM-MIPs (malathion as template and glycidilmethacrylate as pro-hydrophilic co-monomer)	Malathion, terbufos, ethoprophos, phorate, dimethoate, fenamiphos	Honey	0.5–1.9 ng mL^−1^	90.9–97.6	GC-FPD	[84]
2014	Graphene	Dichlorvos, dimethoate, malathion, parathion, parathion	Apple juice	0.04–0.35 ng mL^−1^	69.8–106.2	GC-MS	[76]
2014	MIP (trichlorfon as template)	Trichlorfon	Cucumber, lettuce and radish	4.9 ng g^−1^	77.6–93.2	SPE-CE	[83]
2013	MIP (quinalphos as template)	Diazinon, quinalphos and chlorpyrifos	Apple and grape	0.83–2.8 ng g^−1^	89.7–99.7	HPLC-UV	[104]
2012	MTMOS-TEOS	Chlorpyrifos, diazinon, methidathion, quinalphos, profenofos	Water, red apple, green apple and grape	0.5–0.9 pg mL^−1^	96–111	GC-MS	[20]

**Table 7:** Advantages and limitations of sample preparation techniques commonly used for determination of OPPs.

Extraction Techniques	Advantages	Limitations	Ref.
Liquid-liquid extraction (LLE)	High separation factors High purity products	Time-consuming Large volume of solvent consumption Tedious Formation of emulsion	[27,28]
Solid-Phase Microextraction (SPME)	solvent-free procedure (excludes the use of organic solvents) Miniaturized extraction technique Automated operation reduces the analysis time Feasible to be combined various analytical instruments	Limited sorbent loading Fragile sorbent coating (sorbent easily degrade when exposed to high temperatures) Coating instability in organic solvents	[41,42,44]
Stir-Bar Sorptive Extraction (SBSE)	High sorbent loading effective for extraction of dilute and low concentration samples	incapability to desorb extracted analytes directly into the GC injection port Limited choice of available sorbent	[61,62,63]
Solid-Phase Extraction (SPE)	Numerous sorbents selection Facile procedure High enrichment factor	High cost per sample Susceptible to column blockage	[74,75]

**Table 8:** Advantages and limitations of sorbent materials used in the extraction of OPPs.

Sorbent	Advantages	Limitations	Ref.
IS	High degree of selectivity	High cost Low stability Tedious preparation	[86,87,89]
Graphene	High surface area	Agglomeration of graphene sheets	[75,87,88,92,93,94]
C_18_	Sensitive towards non-polar compounds	Low recovery for polar compounds	[20,95]
MIP	High selectivity Cost effective	Limited to compounds with similar structure	[76,81,85]
Hybrid silica	Porous High surface area Easy to prepare	Require high purity chemicals	[10,20,95,96]

## Data Availability

Not applicable.

## References

[1] Zamani S. (2013). Measurement of pesticides using ultraviolet visible spectrophotometer. Eur. J. Exp. Biol..

[2] Fernandez-Alba A.R., GarcIa-Reyes J.F. (2008). Large-scale multi-residue methods for pesticides and their degradation products in food by advanced LC-MS. Trends Anal. Chem..

[3] Li H., Mehler W.T., Lydy M.J. (2011). Occurrence and distribution of sediment-associated insecticides in urban waterways in the Pearl River Delta, China. Chemosphere.

[4] Chowdhury M.A.Z., Banik S., Uddin B., Moniruzzaman M., Karim N., Gan S.H. (2012). Organophosphorus and carbamate pesticide residues detected in water samples collected from paddy and vegetable fields of the Savar and Dhamrai Upazilas in Bangladesh. Int. J. Environ. Res. Public Health.

[5] De Energia C., Cena A., Paulo U.D.S. (2011). Determination of pesticide residues in tomato using dispersive solid-phase extraction and gas chromatography/ion trap mass spectrometry. J. Braz. Chem. Soc..

[6] Kirchner M., Huskova R., Matisov E., Mocak J. (2008). Fast gas chromatography for pesticide residues analysis using analyte protectants. J. Chromatogr. A.

[7] Trivedi P., Sharma V.P., Srivastava L.P., Malik S. (2014). Multiresidue analysis of organophosphorus pesticides in fruits and vegetables by GC-NPD. Int. J. Adv. Res..

[8] Sun P., Gao Y.L., Xu C., Lian Y.F. (2018). Determination of six organophosphorus pesticides in water samples by three-dimensional graphene. R. Soc. Chem. Adv..

[9] Mehrani Z., Ebrahimzadeh H., Aliakbar A.R., Asgharinezhad A.A. (2018). A poly (4-nitroaniline)/poly (vinyl alcohol) electrospun nanofiber as an efficient nanosorbent for solid phase microextraction of diazinon and chlorpyrifos from water and juice samples. Microchim. Acta.

[10] Nodeh H.R., Wan Ibrahim W.A., Kamboh M.A., Sanagi M.M. (2017). New magnetic graphene-based inorganic-organic sol-gel hybrid nanocomposite for simultaneous analysis of polar and non-polar organophosphorus pesticides from water samples using solid-phase extraction. Chemospere.

[11] Jalili V., Barkhordari A., Ghiasvand A. (2019). New extraction media in microextraction techniques. A review of reviews. Microchem. J..

[12] Samsidar A., Shaarani S., Siddiquee S. (2018). A review of extraction, analytical and advanced methods for determination of pesticides in environment and foodstuffs. Trends Food Sci. Technol..

[13] Sharma B.K. (2006). Environmental Chemistry.

[14] Inoue S., Saito T., Tennefy A.B. (2008). Simultaneous Screening and Detection Methods for Organophosphorus Pesticides and Their Metabolites in Human Biological Samples. Pesticides Research Trends.

[15] Kumar S., Fareedullah M., Sudhakar Y., Venkateswarlu B., Kumar E.A. (2010). Current review on organophosphorus poisoning. Arch. Appl. Sci. Res..

[16] Pore N.E., Pujari K.N., Jadkar S.P. (2011). Organophosphorus poisoning. Int. J. Pharma Biosci..

[17] Girard J.E. (2013). Chapter 17: Insecticides, Herbicides and, Insects Control. Principles of Environmental Chemistry.

[18] Abdel Ghani S.B. (2014). GC-FPD/NPD method development for pesticide multi-residues determination in fresh matrices. Aus. J. Basic Appl. Sci..

[19] Gupta P.K. (2018). Pesticides (agrochemicals). Illustrated Toxicology, with Study Questions.

[20] Wan Ibrahim W.A., Veloo K.V., Sanagi M.M. (2012). Novel sol-gel hybrid methyltrimethoxysilane-tetraethoxysilane as solid phase extraction sorbent for organophosphorus pesticides. J. Chromatogr. A.

[21] (2020). European Communities (EC) Regulation 2020/1085 on Pesticide Regulation. https://ec.europa.eu/food/plant/pesticides/max_residue_levels_en.

[22] (2017). European Communities (EC) Regulation 2017/978 on Pesticide Regulation. https://ec.europa.eu/food/plant/pesticides/max_residue_levels_en.

[23] (2015). European Communities (EC) Regulation 2015/399 on Pesticide Regulation. https://ec.europa.eu/food/plant/pesticides/max_residue_levels_en.

[24] Bylda C., Thiele R., Kobold U., Volmer D.A. (2014). Recent advances in sample preparation techniques to overcome difficulties encountered during quantitative analysis of small molecules from biofluids using LC-MS/MS. Analyst.

[25] Özer E.T., Osman B., Parlak B. (2020). An experimental design approach for the solid phase extraction of some organophosphorus pesticides from water samples with polymeric microbeads. Microchem. J..

[26] Carasek A.E., Mor L. (2018). Basic principles, recent trends and future directions of microextraction techniques for the analysis of aqueous environmental samples. Biochem. Pharmacol..

[27] Clement R.E., Hao C. (2012). Liquid-Liquid Extraction: Basic Principles and Automation. Comprehensive Sampling and Sample Preparation.

[28] Raikos N., Spagou K., Vlachou M., Pouliopoulos A., Thessa E., Tsoukali H. (2009). Development of a liquid-liquid extraction procedure for the analysis of amphetamine in biological specimens by GC-FID. Open For. Sci. J..

[29] Heidari H., Ghanbari-Rad S., Habibi E. (2020). Optimization deep eutectic solvent-based ultrasound-assisted liquid-liquid microextraction by using the desirability function approach for extraction and preconcentration of organophosphorus pesticides from fruit juice samples. J. Food Composit. Anal..

[30] De Oliveira L.L.G., Kudo M.V.F., Lopes C.T., Tarley C.R.T. (2020). Development and multivariate optimization of nanostructured supramolecular liquid-liquid microextraction validated method for highly sensitive determination of methyl parathion in water samples. J. Mol. Liq..

[31] Zacharis C.K., Christophoridis C., Fytianos K. (2012). Vortex-assisted liquid-liquid microextraction combined with gas chromatography-mass spectrometry for the determination of organophosphate pesticides in environmental water samples and wines. J. Sep. Sci..

[32] Rezaee Y.M., Assadi M.R., Millani E., Aghaee F., Ahmadi S. (2006). Berijani. Determination of organic compounds in water using dispersive liquid-liquid microextraction. J. Chromatogr. A.

[33] Zhao E., Zhao W., Han L., Jiang S., Zhou Z. (2007). Application of dispersive liquid-liquid microextraction for the analysis of organophosphorus pesticides in watermelon and cucumber. J. Chromatogr. A.

[34] Herrera-Herrera A.V., Asensio-Ramos M., Hernández-Borges J., Rodríguez-Delgado M.Á. (2010). Dispersive liquid-liquid microextraction for determination of organic analytes. Trends Anal. Chem..

[35] Psillakis E. (2019). Vortex-assisted liquid-liquid microextraction revisited. Trends Anal. Chem..

[36] Musarurwa H., Tavengwa N.T. (2021). Supramolecular solvent-based micro-extraction of pesticides in food and environmental samples. Talanta.

[37] Seebunrueng K., Phosiri P., Apitanagotinon R., Srijaranai S. (2020). A new environment-friendly supramolecular solvent-based liquid phase micro-extraction coupled to high performance liquid chromatography for simultaneous determination of six phenoxy acid herbicides in water and rice samples. Microchem. J..

[38] Salamat Q., Yamini Y., Moradi M., Karimi M., Nazraz M. (2018). Novel generation of nano-structured supramolecular solvents based on an ionic liquid as a green solvent for micro-extraction of some synthetic food dyes. New J. Chem..

[39] Hassan J., Sarkouhi M. (2016). Miniaturised counter current liquid-liquid extraction for organophosphorus pesticides determination. Arab. J. Chem..

[40] Moinfar S., Hosseini M.R.M. (2009). Development of dispersive liquid-liquid microextraction method for the analysis of organophosphorus pesticides in tea. J. Hazard. Mater..

[41] Pawliszyn J., Arthur C.L. (1990). Solid phase microextraction with thermal desorption using fused silica optical fibers. Anal. Chem..

[42] Xu L., Lee H.K. (2012). Sorbent-phase sample preparation in environmental analysis. Pawliszyn.

[43] Picó Y. (2012). Recent advances in sample preparation for pesticides analysis. Comprehensive Sampling and Sample Preparation.

[44] Malik A., McLean M. (2012). Sol-Gel Materials in Analytical Microextraction. Comprehensive Sampling and Sample Preparation.

[45] Whang C., Pawliszyn J. (1998). Solid phase microextraction coupled to capillary electrophoresis. Anal. Commun..

[46] Eisert R., Pawliszyn J. (1997). Design of automated solid-phase microextraction for trace analysis of organic compounds in aqueous samples. J. Chromatogr..

[47] Arthur C.L., Killam L.M., Buchholz K.D., Pawliszyn J., Berg J.R. (1992). Automation and optimisation of solid-phase microextraction. Anal. Chem..

[48] Blasco C., Fernández M., Picó Y., Font G. (2004). Comparison of solid-phase microextraction and stir bar sorptive extraction for determining sixorganophosphorus insecticides in honey by liquid chromatography–mass spectrometry. J. Chromatogr. A.

[49] Rodrigues F.D., Mesquita P.R.R., de Oliveira L.S., de Oliveira F.S., Menezes A., Pereira P.A.D., de Andrade J.B. (2011). Development of a headspace solid-phase microextraction/gas chromatography-mass spectrometry method for determination of organophosphorus pesticide residues in cow milk. Microchem. J..

[50] Jabbari M., Razmi H., Farrokhzadeh S. (2016). Application of magnetic graphene nanoparticles for determination of organophosphorus pesticides using solid-phase microextraction. Chromatographia.

[51] Mardanbeigi D., Ebrahimi M. (2015). Fe_3_O_4_@Graphene nanocomposite reinforced hollow fiber-solid phase microextraction for preconcentration and determination of organophosphate pesticide in environmental samples. Entomol. Appl. Sci. Lett..

[52] Saraji M., Jafari M.T., Mossaddegh M. (2017). Chemically modified halloysite nanotubes as a solid-phase microextraction coating. Anal. Chim. Acta.

[53] Wu R., Qian X., Rui X., Liu H., Yadian B., Zhou K., Wei J., Yan Q., Feng X.Q., Long Y. (2014). Zeolitic imidazolate framework 67-derived high symmetric porous Co_3_O_4_ hollow dodecahedra with highly enhanced lithium storage capability. Small.

[54] Chaikittisilp W., Ariga K., Yamauchi Y. (2013). A new family of carbon materials: Synthesis of MOF-derived nanoporous carbons and their promising applications. J. Mater. Chem. A.

[55] Pang Y., Zang X., Li H., Liu J., Chang Q., Zhang S., Wang C., Wang Z. (2020). Solid-phase microextraction of organophosphorous pesticides from food samples with a nitrogen-doped porous carbon derived from g-C3N4 templated MOF as the fiber coating. J. Hazard. Mater..

[56] Wang D., Wang Y., Chen Y., Liu W., Wang H., Zhao P., Li Y., Zhang J., Dong Y., Hu S. (2018). Coal tar pitch derived N-doped porous carbon nanosheets by the in-situ formed g-C_3_N_4_ as a template for supercapacitor electrodes. Electrochim. Acta.

[57] Gu W., Hu L., Li J., Wang E. (2016). Hybrid of g-C_3_N_4_ assisted metal-organic frameworks and their derived high-efficiency oxygen reduction electrocatalyst in the whole pH range. ACS Appl. Mater. Interface.

[58] Bao W., Liu L., Wang C., Choi S., Wang D., Wang G. (2018). Facile synthesis of crumpled nitrogen-doped MXene manosheets as a new sulfur host for lithium-sulfur batteries. Adv. Energy Mater..

[59] Ruiz del Castillo M.L., Rodríguez-Valenciano M., Flores G., Blanch G.P. (2019). New method based on Solid Phase Microextraction and Multidimensional gas chromatography-mass spectrometry to determine pesticides in strawberry jam. Food Sci. Technol..

[60] Ghavidel F., Shahtaheri S.J., Jazani R.K., Torabbeigi M., Froushani A.R., Khadem M. (2014). Optimisation of solid phase microextraction procedure followed by gas chromatography with electron capture detector for pesticides butachlor and chlorpyrifos. Am. J. Anal. Chem..

[61] Abdulra’uf L., Tan G.H. (2014). Review of SBSE technique for the analysis of pesticide residues in fruits and vegetables. Chromatographia.

[62] Baltussen E., Cramers C.A., Sandra P.J.F. (2002). Sorptive sample preparation: A review. Anal. Bioanal. Chem..

[63] Sa´nchez-Rojas F., Bosch-Ojeda C., Cano-Pavon J.M. (2009). A review of stir-bar sorptive extraction. Chromatographia.

[64] Jafari M.T., Rezaei B., Bahrami H. (2018). Talanta Zirconium dioxide-reduced graphene oxide nanocomposite-coated stir-bar sorptive extraction coupled with ion mobility spectrometry for determining ethion. Talanta.

[65] Ahmadkhaniha R., Rastkari N. (2016). Development of a carbon nanotube-coated stir bar for determination of organophosphorus pesticides in water. Asia-Pac. J. Chem. Eng..

[66] Yu C., Hu B. (2009). Sol-gel polydimethylsiloxane/poly(vinylalcohol)-coated stir bar sorptive extraction of organophosphorus pesticides in honey and their determination by large volume injection GC. J. Sep. Sci..

[67] Ahmadkhaniha R., Rastkari N. (2013). Development of a New Coating for Sorptive Extraction by Stir Bars. WIPO/PCT.

[68] Wan Ibrahim W.A., Wan Ismail W.N., Abdul Keyon A.S., Sanagi M.M. (2011). Preparation and characterization of a new sol-gel hybrid based tetraethoxysilane-polydimethylsiloxane as a stir-bar extraction sorbent materials. J. Sol-Gel Sci. Technol..

[69] Benedé J.L., Chisvert D.L.A., Giokas A. (2014). Salvador, Development of stir bar sorptive-dispersive microextraction mediated by magnetic nanoparticles and its analytical application to the determination of hydrophobic organic compounds in aqueous media. J. Chromatogr. A.

[70] Madej K., Jonda A., Borcuch A., Piekoszewski W., Chmielarz L., Gil B. (2019). A novel stir bar sorptive-dispersive microextraction in combination with magnetically modified graphene for isolation of seven pesticides from water samples. Microchem. J..

[71] Hasan C.K., Ghiasvand A., Lewis T.W., Nesterenko P.N., Paull B. (2020). Recent advances in stir-bar sorptive extraction: Coatings, technical improvements, and applications. Anal. Chim. Acta.

[72] Ansari S., Talebpour Z., Molaabasi F., Bijanzadeh H.R., Khazaeli S. (2016). Quantitative 31P NMR for simultaneous trace analysis of organophosphorus pesticides in aqueous media using the stir bar sorptive extraction method. J. Appl. Spectrosc..

[73] Hu C., He M., Chen B., Hu B. (2013). A sol-gel polydimethylsiloxane/ polythiophene coated stir bar sorptive extraction combined with gas chromatography-flame photometric detection for the determination of organophosphorus pesticides in environmental water samples. J. Chromatogr. A.

[74] Biziuk M., Zwir-Ferenc A. (2006). Solid phase extraction technique: Trends, opportunities and applications. Polish J. Environ. Stud..

[75] Płotka-Wasylka J., Szczepa´nska N., Guardia de la M., Namie´snik J. (2015). Miniaturized solid-phase extraction techniques. Trends Anal. Chem..

[76] Han Q., Wang Z., Xia J., Zhang X., Wang H., Ding M. (2014). Application of graphene for the SPE clean-up of organophosphorus pesticides residues from apple juices. J. Sep. Sci..

[77] Maddah B., Hasanzadeh M. (2017). Fe_3_O_4_/CNT magnetic nanocomposites as adsorbents to remove organophosphorus pesticides from environmental water. Int. J. Nanosci. Nanotechnol..

[78] Pellicer-Castell E., Belenguer-Sapiña C., Amorós P., El Haskouri J., Herrero-Martínez J.M., Mauri-Aucejo A. (2018). Study of silica-structured materials as sorbents for organophosphorus pesticides determination in environmental water samples. Talanta.

[79] Xie J., Liu T., Song G. (2013). Simultaneous analysis of organophosphorus pesticides in water by magnetic solid-phase extraction coupled with GC-MS. Chromatographia.

[80] Ng N.T., Kamaruddin A.F., Ibrahim W.A.W., Sanagi M.M., Keyon A.S.A. (2018). Advances in organic-inorganic hybrid sorbents for the extraction of organic and inorganic pollutants in different types of food and environmental samples. J. Sep. Sci..

[81] Buszewski B., Szultka M. (2012). Past, present, and future of solid phase extraction: A review. Crit. Rev. Anal. Chem..

[82] Boulanouar S., Mezzache S., Combès A., Pichon V. (2018). Molecularly imprinted polymers for the determination of organophosphorus pesticides in complex samples. Talanta.

[83] Zhao T., Gao H., Wang X., Zhang L., Qiao X., Xu Z. (2014). Study on a molecularly imprinted solid-phase extraction coupled to capillary electrophoresis method for the determination of trace trichlorfon in vegetables. Food Anal. Methods.

[84] He J., Song L., Chen S., Li Y., Wei H., Zhao D., Gu K. (2015). Novel restricted access materials combined to molecularly imprinted polymers for selective solid-phase extraction of organophosphorus pesticides from honey. Food Chem..

[85] Bures P., Huang Y., Oral E., Peppas N.A. (2001). Surface modifications and molecular imprinting of polymers in medical and pharmaceutical applications. J. Control. Release.

[86] Sadilek P., Satinsky D., Solich P. (2007). Using restricted-access materials and column switching in high-performance liquid chromatography for direct analysis analysis of biologically-active compounds in complex matrices. Trends Anal. Chem..

[87] Hennion M.C., Pichon V. (2013). Immuno-based sample preparation for trace analysis. J. Chromatogr. A.

[88] Xu Z., Deng H., Lei H., Jiang Y., Campbell K., Shen Y., Sun Y. (2012). Development of a broad-specificity monoclonal antibody-based immunoaffinity chromatography cleanup for organophosphorus pesticide determination in environmental samples. J. Agric. Food Chem..

[89] Pichon V., Bouzige M., Mieége C., Hennion M.C. (1999). Immunosorbents: Natural molecular recognition materials for sample preparation of complex environmental matrices. Trends Anal. Chem..

[90] Chen L., Zhang X., Xu Y., Du X., Sun X., Sun L. (2010). Determination of fluoroquinolone antibiotics in environmentalwater samples based on magnetic molecularly imprinted polymer extraction followed by liquid chromatography-tandem mass spectrometry. Anal. Chim. Acta.

[91] Akerdi A.G., Bahrami S.H., Arami M., Pajootan E. (2016). Photocatalytic discoloration of acid red 14 aqueous solution using titania nanoparticles immobilized on graphene oxide fabricated plate. Chemosphere.

[92] Mahpishanian S., Sereshti H. (2016). Three-dimensional graphene aerogel-supported iron oxide nanoparticles as an efficient adsorbent for magnetic solid phase extraction of organophosphorus pesticide residues in fruit juices followed by gas chromatographic determination. J. Chromatogr. A.

[93] Liu Q., Shi J., Zeng L., Wang T., Cai Y., Jiang G. (2014). Evaluation of grapheme as an advantageous adsorbent for solid-phase extraction with chlorophenols as model analytes. J. Chromatogr. A.

[94] Andrade-Eiroa A., Canle M., Leroy-Cancellieri V., Cerdà V. (2016). Solid-phase extraction of organic compounds: A critical review (Part I). Trends Anal. Chem..

[95] Wan Ibrahim W.A., Wan Ismail W.N., Sanagi M.M. (2013). Selective and simultaneous solid phase extraction of polar and non-polar organophosphorus pesticides using sol-gel hybrid silica-based sorbent. J. Teknol..

[96] Veloo K.V., Ibrahim N.A.S. (2020). Solid-phase extraction using chloropropyl functionalized sol-gel hybrid sorbent for simultaneous determination of organophosphorus pesticides in selected fruit samples. J. Separat. Sci..

[97] Michelangelo A., Lehotay S.J. (2003). Fast and easy multiresidue method employing acetonitrile extraction/partitioning and “dispersive solid-phase extraction for the determination of pesticide residues in produce. J. AOAC Int..

[98] Fakhari A.R., Aladaghlo Z. (2019). Development of a new solvent-assisted dispersive solid-phase extraction followed by ion mobility spectrometry for trace determination of organophosphorus pesticides in environmental water samples. Sep. Sci. Plus.

[99] Amiri A., Baghayeri M., Vahdati-Nasab N. (2020). Effective extraction of organophosphorus pesticides using sol–gel based coated stainless steel mesh as novel solid-phase extraction sorbent. J. Chromatogr. A.

[100] Amiri F. (2017). Ghaemi, Graphene grown on stainless steel mesh as a highly effi- cient sorbent for sorptive microextraction of polycyclic aromatic hydrocarbons from water samples. Anal. Chim. Acta.

[101] Li D., He M., Chen B., Hu B. (2019). Metal organic frameworks-derived magnetic nanoporous carbon for pre-concentration of organophosphorus pesticides from fruit samples followed by gas chromatography-flame photometric detection. J. Chromatogr. A.

[102] Meseguer-Lloret S., Torres-Cartas S., Catala-Icardo M., Simo-Alfonso E.F., Herrero-Martïnez J.M. (2017). Extraction and preconcentration of organophosphorus pesticides in water by using a polymethacrylate-based sorbent modified with magnetic nanoparticles. Anal. Bioanal. Chem..

[103] Korrani Z.S., Ibrahim W.A.W., Nodeh H.R., Aboul-Enein H.Y., Sanagi M.M. (2016). Simultaneous preconcentration of polar and non-polar organophosphorus pesticides from water samples by using a new sorbent based on mesoporous silica. J. Sep. Sci..

[104] Sanagi M.M., Salleh S., Wan Ibrahim W.I., Abu A. (2013). Molecularly imprinted polymer solid-phase extraction for the analysis of organophosphorus pesticides in fruit samples. J. Food Composit. Anal..

